# Increased Cytokeratin 19 Fragment Levels Are Positively Correlated with Adenosine Deaminase Activity in Malignant Pleural Effusions from Adenocarcinomas

**DOI:** 10.1155/2018/2609767

**Published:** 2018-05-08

**Authors:** Jorge Luiz Barillo, Cyro Teixeira da Silva Junior, Patricia Siqueira Silva, Joeber Bernardo Soares de Souza, Salim Kanaan, Analucia Rampazzo Xavier, Elizabeth Giestal de Araujo

**Affiliations:** ^1^Antonio Pedro University Hospital, Fluminense Federal University, Niteroi, RJ, Brazil; ^2^General Hospital Santa Teresa, Petropolis, RJ, Brazil; ^3^Department of Clinics, School of Medicine, Fluminense Federal University, Niteroi, RJ, Brazil; ^4^Clinical Analysis Postgraduate Program, Fluminense Federal University, Niteroi, RJ, Brazil; ^5^Department of Clinical Pathology, School of Medicine, Fluminense Federal University, Niteroi, RJ, Brazil; ^6^Department of Neurobiology, Biology Institute, Fluminense Federal University, Niteroi, RJ, Brazil

## Abstract

Adenosine deaminase (ADA) and cytokeratin 19 (CK19) are known pleural biomarkers. Although ADA in humans functions mainly in the immune system, it also appears to be associated with the differentiation of epithelial cells. Keratin filaments are important structural stabilizers of epithelial cells and potent biomarkers in epithelial differentiation. This study aimed to investigate the simultaneous presence of the ADA enzyme and CK19 fragments to assess epithelial differentiation in malignant and benign pleural fluids. Diagnosis of the cause of pleural effusion syndrome was confirmed by means of standard examinations and appropriate surgical procedures. An ADA assay, in which ADA irreversibly catalyzes the conversion of adenosine into inosine, was performed using a commercial kit. The CK19 assay was performed using a CYFRA 21-1 kit, developed to detect quantitative soluble fragments of CK19 using an electrochemiluminescence immunoassay. One hundred nineteen pleural fluid samples were collected from untreated individuals with pleural effusion syndrome due to several causes. ADA levels only correlated with CK19 fragments in adenocarcinomas, with high significance and good correlation (rho = 0.5145, *P* = 0.0036). However, further studies are required to understand this strong association on epithelial differentiation in metastatic pleural fluids from adenocarcinomas.

## 1. Introduction

Metastatic diseases are a predominant cause of pleural effusions in lung cancer. A malignant pleural effusion (MPE) may be the initial signal of lung cancer in 10–50% of patients with stage IV disease, according to the tumor, node, and metastasis (TNM) staging system [1]. Carcinomas of the lung and breast and lymphomas account for approximately 75% of MPE cases [1]. Lung epithelial cancer types, such as adenocarcinoma, squamous-cell carcinoma, and neuroendocrine tumors, are associated with the highest levels of biomarkers in serum and pleural fluid [2]. Cancer biomarkers are substances that are usually secreted by malignant cells or by the host in response to a tumor [3].

Adenosine deaminase (ADA) is a biomarker of pleural tuberculosis. However, the main function of this enzyme is to trigger the immune system in humans. Moreover, ADA also appears to be associated with neurotransmission, gestation, and the differentiation of epithelial cells [4]. Cytokeratins (CKs) are cancer biomarkers and are the main structural elements of the cytoskeleton in epithelial cells. CK19 is expressed in the epithelium covering the bronchial tree and is overexpressed in lung cancer [5]. Conflicting information on the relationship between ADA and cancer has been reported in previous studies, some of which have found ADA activity to be increased in malignant tissues and others of which have found it to be decreased [6–8].

ADA (E.C. 3.5.4.4) is an enzyme of the purine pathway that catalyzes the deamination of adenosine and 2′-deoxyadenosine into inosine and 2′-deoxyinosine, respectively. The conversion of inosine leads to hypoxanthine and uric acid or other mononucleosides. ADA is intracellularly expressed on the cell membrane. It does not have its own transmembrane domain and is therefore associated with a surface glycoprotein with two molecules of dipeptidyl peptidase IV, called CD 26. ADA is released by growth factors and cytokines, such as IL-2, IL-12, and interferon-gamma, and exhibits increased levels during malignancy [4].

CKs constitute an intermediate filament group that represents more than 20 different types of polypeptides, which are the major components of the cytoskeleton, a proteinaceous structural framework within the cellular cytoplasm. They are expressed in various sets, according to the epithelial type and the degree of differentiation. In normal human cells, CKs are classified as acidic type I (CK9–CK23) or neutral-basic type II (CK1–CK8). CK19 belongs to the class of type I cytokeratins. In cancer diagnosis, keratins are useful as diagnostic and prognostic biomarkers, active regulators of epithelial tumorigenesis, and indicators of treatment responsiveness [9–13]. Cytokeratin 19 fragments (CK19) are expressed in almost all epithelial malignancies, including breast cancer, lung cancer, colorectal carcinoma, cervical carcinoma, and papillary thyroid carcinoma. Its main functions are the maintenance of epithelial cell integrity, the mediation of stress responses, cell signaling, apoptosis, and participation in the immune response, including extravasation, migration, signaling, antigen recognition, phagocytosis, and cellular activation [9–13]. We hypothesized that a relationship could exist between pleural ADA and CK19 in patients with malignant pleural effusion, which has not been previously reported. Therefore, the objective of our study was to investigate the simultaneous occurrence of ADA and CK19 in malignant pleural fluids.

## 2. Materials and Methods

### 2.1. Design and Study Population

This was a prospective study conducted from January 2014 to January 2016 at Antonio Pedro Hospital, a teaching center of Fluminense Federal University, located in Niteroi, and General Hospital Santa Teresa, located in Petropolis, both in the State of Rio de Janeiro, Brazil. The Antonio Pedro Hospital Ethics Committee approved this study under the number 80/02, according to the guidelines of the Helsinki Declaration. Written consent was obtained from all patients.

### 2.2. Inclusion and Exclusion Criteria for Patients in the Study

Pleural fluid samples were collected from continuous untreated individuals with pleural effusion syndrome due to several causes. The diagnosis of the cause of pleural effusion syndrome (PES) was confirmed by means of standard examinations and the use of appropriate surgical procedures [14]. The first biochemical tests used to diagnose a pleural transudate or exudate were current criteria for the dosage of total proteins and lactate dehydrogenase only in pleural fluids [15]. When a causal diagnosis of PES was unconfirmed after a thoracentesis procedure with laboratory evaluation of the pleural fluid, a closed-needle pleural biopsy was performed using Cope's needle. If PES persisted and symptoms increased or when it was not possible to differentiate malignancy and tuberculosis, the patient was referred for video-assisted thoracoscopic surgery [14]. The exclusion criteria included absolute contraindications, refusal to accept thoracentesis or other invasive procedures, use of immunosuppressive medications, hemolysis in pleural liquids, renal failure, HIV infection, and pleural effusion of an unknown cause. Patients with serum levels of bilirubin greater than 65 mg/dL, lipid content great than 1500 mg/dL, and a rheumatoid factor concentration great than 1500 IU/M were excluded from our study because these factors interfere with biomarker levels [16, 17].

### 2.3. ADA Assay

ADA assays, in which ADA irreversibly catalyzes the conversion of adenosine into inosine, with hydrogen peroxide produced in the final enzymatic reaction, were performed using a commercial kit. The assay is ready to use for automated chemistry analyzers using the kinetic method. Its principle relies on the detection of H_2_O_2_, and it is more sensitive than the colorimetric method of Giusti and Galanti. The assay is based on the Berthelot reaction, in which a blue dye produced by phenol–sodium hypochlorite is used to analyze the concentration of ammonia in pleural fluids. One unit of ADA is defined as the amount of ADA that generates one *μ*mol of inosine from adenosine per min at 37°C [16].

### 2.4. Cytokeratin 19 Assay

The assay was performed using a CYFRA 21-1 kit designed to quantify soluble CK19 using an electrochemiluminescence immunoassay (ECLIA). The cut-off limit of the ECLIA in serum and plasma is 3.3 ng/mL with a specificity of 95%. The assay has the ability to detect the tris(2,2′-bipyridyl) ruthenium complex, an electrochemically luminescent molecule. The technique can be used for sandwich format and competitive immunoassays. CYFRA 21-1 is identified by two specific mouse monoclonal antibodies (Ks 19-1 and BM 19-21). These antibodies are directed toward two different antigenic determinants of a fragment of CK19. The sandwich complex binds to the particulate solid phase through the interaction of biotin and streptavidin. The reaction mixture is then aspirated into the measuring cell. The microparticles are magnetically captured on the electrode. The chemiluminescence emission is induced by applying voltage to the electrode and is measured by a photomultiplier [17]. The pleural fluids for the CYFRA 21-1 and ADA assays were stored at −20°C. CYFRA 21-1 and ADA in the pleural fluid were determined in a blinded manner, without information on the definitive diagnosis.

### 2.5. Statistical Approach

Both descriptive and inferential statistics were calculated using GraphPad (GraphPad Software Inc., version 6.0, La Jolla, CA, USA). The laboratory data were analyzed by means of a univariate analysis. A *P* value less than 0.05 determined from a two-tailed test was considered statistically significant, to reject the null hypothesis with 5% probability of a type I error. The Shapiro–Wilk test was used to assess the normality of the data. The quantitative variables were assumed to be normally distributed and expressed in terms of their means and standard deviations (SDs), and those with nonnormal distributions were expressed in terms of their medians and interquartile ranges (IQRs). Qualitative or categorical variables were expressed in terms of proportions. To compare the information on MPE and benign pleural effusions (BPEs) between the two groups, data were analyzed using Student's *t*-test if the data were normally distributed or using the Mann–Whitney *U* test if the data did not follow a normal distribution. The chi-squared test was used to compare proportions, as recommended by Campbell (2007) and Richardson (2011). The Kruskal–Wallis *H* test (KW test) and post hoc Dunn test were used to compare ADA and CK19 levels in the adenocarcinoma group versus three or more unpaired (independent) samples in the several groups studied. The correlations between the ADA and CK19 results and several causes of pleural effusion were calculated using Spearman's rank correlation coefficient rho, which is commonly used for nonparametric correlation. By convention, correlation coefficients above 0.70 represent strong correlation, values between 0.50 and 0.70 represent good correlation, values between 0.30 and 0.50 represent moderate correlation, and values less than 0.30 represent weak correlation [18].

## 3. Results

The sample size used in this study was 119 pleural fluid samples from untreated individuals with pleural effusion syndrome due to several causes. The demographic characteristics of the patients are summarized in Table 1. In comparison to those in the BPE group, the CK19 fragment levels in the MPE group were significantly increased (median level: 85.03 ng/mL versus 13.41 ng/mL, *P* = 0.0001).

The patients were separated into eight subgroups of benign and malignant pleural fluids (Table 2): adenocarcinomas of different sites (*n* = 30), tuberculosis (*n* = 28), transudates by congestive heart failure (*n* = 21), simple and complicated parapneumonic effusion (*n* = 15), nontuberculous empyema (*n* = 8), squamous-cell carcinomas of different sites (*n* = 7), lymphomas (*n* = 4), and other causes (*n* = 6), including Dressler syndrome (*n* = 3), melanocarcinoma (*n* = 1), chylothorax (*n* = 1), and leukemia (*n* = 1). Differences in the medians of the ADA and CK19 levels in the several groups of pleural fluid were statistically significant (Kruskal–Wallis test, *P* < 0.0001), as shown in Table 2.

ADA levels exhibited a significant *P* value (Dunn's test, *P* < 0.05) in patients with tuberculosis versus adenocarcinoma, as well as parapneumonic and transudative pleural fluid. However, the *P* value according to Dunn's test was not significant (*P* > 0.05) for tuberculosis versus other pleural fluids: mainly empyemas, lymphomas, and squamous-cell carcinomas (Table 2).

The CK19 analysis helped us to exclude adenocarcinomas versus tuberculosis, transudates, and parapneumonic effusions with a highly significant *P* value (Dunn's test, *P* < 0.0001), as shown in Table 2. For the remaining pleural fluids, the *P* value with Dunn's test was not significant (*P* > 0.05).

**Figure 1:**
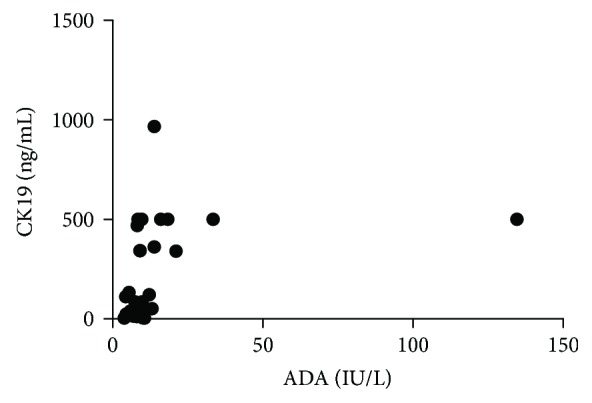
Spearman's correlation between CK19 fragments (ng/mL) and ADA levels (IU/L) in adenocarcinoma groups (*n* = 30, rho = 0.5145, *P* = 0.0036).

Finally, we determined the value of Spearman's correlation coefficient between adenosine deaminase and cytokeratin 19 fragments in the pleural fluids of the 119 patients with final diagnoses. As indicated in Table 3, ADA levels were significantly positively correlated with CK19 fragments in adenocarcinomas (rho = 0.5145, *P* = 0.0036, Figure 1) and significantly negatively correlated with other diseases (rho = −0.9429, *P* = 0.0167). No significant correlations were found with tuberculosis (rho = −0.9614, *P* = 0.6265), transudates (rho = 0.1805, *P* = 0.4336), parapneumonics (rho = −0.1321, *P* = 0.6387), empyemas (rho = −0.5663, *P* = 0.1511), squamous-cell carcinomas (rho = 0.0714, *P* = 0.9063), or lymphomas (rho = 0.4000, *P* = 0.7500).

## 4. Discussion

Malignant pleural fluids may be useful in modeling the hierarchical progression and heterogeneity of lung cancer [19]. The results of the present study provide the first evidence that increased levels of CK19 in pleural fluids are associated with adenosine deaminase activity in malignant pleural effusions from adenocarcinomas.

A paper recently published by our group described a study in which patients with benign pleural effusions were predominantly male [20]. The median and range of patients' ages in that study were similar to those shown in Table 1. Tuberculosis and adenocarcinomas are the most frequent causes of exudative pleural effusions in Brazil (Table 2). Table 2 shows the determination of cytokeratin 19 fragments and ADA activity in the pleural fluid of the patients in this study presenting with confirmed pleural effusion syndrome from any of several origins, mainly adenocarcinoma and other diseases such as tuberculosis. ADA is undoubtedly the best biomarker for pleural tuberculosis in clinical practice [20]. Our study, similar to many others, shows that the ADA level is useful in the differential diagnosis between tuberculosis and adenocarcinoma in pleural fluids. As shown in Table 2, the CYFRA 21-1 assay detected circulating fragments of cytokeratin 19 in adenocarcinomas and squamous-cell carcinomas, and the difference between pleural tuberculosis and other diseases was significant (KW = 37.19, *P* < 0.0001). In clinical practice, CK19 is a useful biomarker for the diagnosis of lung cancer [21]. However, it is important to remember that diagnostic decisions regarding MPE should depend on conclusive cytology results for pleural fluid or pleural biopsy [22].

We attempted to explore the possible causes for the high levels of CK19 in lung cancer. Human lung cancer cells in pleural fluids have been found to be more invasive than cancer cells from primary sites. This difference may be related to epithelial–mesenchymal transition (EMT). EMT is reactivated during adulthood under conditions of disease, such as cancer. EMT determines the severity of cancer and contributes to metastasis [5]. EMT is a progressive malignant cell program that enhances the mobility, invasion, resistance to apoptotic stimuli, and therapeutic resistance of cancer cells [23]. Levels of CK19 may reflect cytoskeleton formation in malignant cells and the association with the degree of cancer differentiation toward the squamous epithelium. The keratin content increases when epithelial cells transform into cancer cells. Soluble fragments of CK19 are released into the pleural fluid as a result of necrosis or apoptosis of malignant cells with increased caspase-3 activity [24].

Spearman's correlation coefficients obtained for the correlations between adenosine deaminase and cytokeratin 19 fragments in pleural fluids were significantly positive for adenocarcinomas and significantly negative for a heterogeneous group of other diseases, including Dressler syndrome, melanocarcinoma, chylothorax, and leukemia, as shown in Table 3. These findings indicate a highly significant relationship between these two biomarkers, mainly in the pleural fluids from adenocarcinomas (Figure 1). There was a good correlation or relationship between ADA and CK19 in pleural adenocarcinomas (*n* = 30): rho = 0.5145 and *P* = 0.0036 (Table 3). There is a tendency to believe that a correlation between biomarkers means that one causes or influences changes in the other. However, a correlation does not imply causation, and an unknown third factor can be responsible for fluctuations in both ADA and CK19 in pleural adenocarcinoma cells. Furthermore, what is the explanation for the significant correlation between the levels of ADA and CK19 fragments in pleural adenocarcinoma? We could speculate that cancer cells contain high levels of extracellular adenosine. When binding to its receptors, the extracellular adenosine exhibits angiogenic and anti-inflammatory actions. It also exhibits intracellular action that modulates purine levels and affects tumor growth and metastatic phenotype [25]. Therefore, a need for increased adenosine in adenocarcinoma cells leads to increased ADA production (Table 3).

Limitations of the study. The interpretation of this study have been considered in relation to the controversial results in the literature to elucidate the usefulness of tumor biomarkers in malignant pleural effusions when the site of primary cancer is unknown [26, 27]. However, the use of a biomarker for diagnosis always limits its result interpretation on a previous analysis of clinical manifestations, image findings, epidemiological profile of the evaluated patient, and a false-positive level for other possible diseases [20].

## 5. Conclusions

The results of our study demonstrate a strong association between ADA and CK19 levels in pleural metastatic adenocarcinomas. However, further studies are required to fully understand whether adenosine deaminase and cytokeratin 19 fragments play the same important role in epithelial differentiation in metastatic pleural fluids from adenocarcinomas.

## Figures and Tables

**Table 1:** Demographic characteristics, ADA levels, and CK19 of controls (benign pleural effusions) and malignant pleural effusions (cases) in 119 patients.

Demographic characteristics and pleural biomarkers	MPE (*n* = 43)	BPE (*n* = 76)	Test (*P* value)
Male, *n* (%)	15 (35.0)	36 (47.0)	Chi-squared = 0.608 (*P* = 0.4354)
Female, *n* (%)	28 (65.0)	40 (53.0)	Chi-squared = 0.959 (*P* = 0.3275)
Mean age (years ± SD)	62.33 ± 12.37	60.95 ± 21.37	*T* = 1.323 (*P* = 0.1931)
Median total ADA levels (IU/L) in normal scale (IQR)	9.76 (6.74–13.90)	11.63 (6.64–34.32)	*U* = 1407.0 (*P* = 0.2102)
Median CK19 levels (ng/mL) in normal scale (IQR)	85.03 (24.61–362.3)	13.41 (7.06–32.30)	*U* = 586.0 (*P* = 0.0001)

Abbreviations: MPE: malignant pleural effusion; BPE: benign pleural effusion; SD: standard deviation; ADA: adenosine deaminase; CK19: cytokeratin 19 fragments; IQR: interquartile range, 25th–75th percentiles.

**Table 2:** Adenosine deaminase and cytokeratin 19 fragment levels in the pleural fluids of 119 patients with final diagnoses.

Diseases	Patients (*n*)	Adenosine deaminase, median (IU/L) (IQR)	Cytokeratin 19 fragments, median (ng/mL) (IQR)
Adenocarcinomas	30	9.41 (6.56–13.31)	98.96 (23.76–476.6)
Tuberculosis	28	39.08 (26.66–45.96)	16.72 (8.58–34.72)
Transudates	21	3.26 (2.09–7.73)	8.40 (5.91–13.94)
Parapneumonics	15	9.38 (5.68–9.97)	12.09 (6.06–28.86)
Empyemas	08	32.94 (16.07–61.70)	24.55 (2.03–205.3)
Squamous cell carcinomas	07	13.11 (11.08–18.65)	52.11 (24.63–236.2)
Lymphomas	04	21.64 (10.0–750.9)	14.99 (3.96–60.86)
Other	06	10.14 (5.26–24.30)	27.43 (14.61–32.40)

Abbreviations: IQR: interquartile range; ADA: adenosine deaminase; CK19: cytokeratin 19 fragments. Other diseases: Dressler syndrome (*n* = 3), melanocarcinoma (*n* = 1), chylothorax (*n* = 1), and leukemia (*n* = 1). Kruskal–Wallis test: *H* = 63.10 for ADA (*P* < 0.0001) with *P* < 0.05 for tuberculosis versus adenocarcinoma, parapneumonic, and transudate; *P* > 0.05 for tuberculosis versus other groups. For CK19, *H* = 37.19 (*P* < 0.0001) with *P* < 0.05 for adenocarcinoma versus tuberculosis, transudate, and parapneumonic. Diseases remaining: *P* > 0.05.

**Table 3:** Spearman's rank correlation coefficients (rho) between adenosine deaminase and cytokeratin 19 fragments in the pleural fluids of 119 patients with final diagnoses.

Diseases	Rho	95% confidence intervals	*P* value (two tailed)
Adenocarcinomas	0.5145	0.178–0.743	0.0036
Tuberculosis	−0.9614	−0.466 to 0.297	0.6265
Transudates	0.1805	−0.285 to 0.577	0.4336
Parapneumonics	−0.1321	−0.614 to 0.421	0.6387
Empyemas	−0.5663	−0.908 to 0.230	0.1511
Squamous cell carcinomas	0.07143	−0.720 to 0.782	0.9063
Lymphomas	0.4000	−0.911 to 0.983	0.7500
Other	−0.9429	−0.993 to 0.560	0.0167

Interpretation of rho: values above 0.70, strong correlation; 0.50–0.70, good correlation; 0.3–0.5, moderate correlation; and less than 0.30, poor correlation.
